# Wearable Sensors Integrated with Virtual Reality: A Self-Guided Healthcare System Measuring Shoulder Joint Mobility for Frozen Shoulder

**DOI:** 10.1155/2019/7681237

**Published:** 2019-04-10

**Authors:** Jianjun Cui, Shih-Ching Yeh, Si-Huei Lee

**Affiliations:** ^1^Fudan University, Shanghai 200433, China; ^2^Taipei Veterans General Hospital, Taipei 11217, Taiwan

## Abstract

Frozen shoulder is a common clinical shoulder condition. Measuring the degree of shoulder joint movement is crucial to the rehabilitation process. Such measurements can be used to evaluate the severity of patients' condition, establish rehabilitation goals and appropriate activity difficulty levels, and understand the effects of rehabilitation. Currently, measurements of the shoulder joint movement degree are typically conducted by therapists using a protractor. However, along with the growth of telerehabilitation, measuring the shoulder joint mobility on patients' own at home will be needed. In this study, wireless inertial sensors were combined with the virtual reality interactive technology to provide an innovative shoulder joint mobility self-measurement system that can enable patients to measure their performance of four shoulder joint movements on their own at home. Pilot clinical trials were conducted with 25 patients to confirm the feasibility of the system. In addition, the results of correlation and differential analyses compared with the results of traditional measurement methods exhibited a high correlation, verifying the accuracy of the proposed system. Moreover, according to interviews with patients, they are confident in their ability to measure shoulder joint mobility themselves.

## 1. Introduction

Frozen shoulder is the common name for impaired shoulder movement caused by injury to the shoulder capsule and soft tissues. Clinically, frozen shoulder is a common shoulder condition. The symptoms of this condition include restriction of active or passive joint motion, stiffness, aching, and loss of muscular strength in the shoulder. Such symptoms typically manifest for 2 years [1–5], although in severe cases, they can persist for more than 5 years. Generally, patients cannot move their shoulder because of injury to the soft tissue surrounding the shoulder. The accompanying pain causes them to avoid all movements, which further exacerbates the condition. The prevalence of frozen shoulder in the general population is between 2% and 5%. This condition is typically experienced between the ages of 40 and 65 years, with a higher occurrence rate for women compared with that for men (at a ratio of 58 : 42) [6]. People diagnosed with diseases such as diabetes and hyperthyroidism also exhibit a higher than average occurrence rate [7].

Regarding medication treatments, medicines such as Panadol and nonsteroid anti-inflammatory drugs can be considered. In addition, steroid injections into the shoulder joint can also be administered to ease pain. Medication treatments are generally provided to alleviate pain and reduce inflammation. However, for certain patients with severe symptoms, the effect of such treatments is limited. Therefore, alternative physical therapies are necessary to effectively restore shoulder joint function [8]. Measurements of shoulder joint mobility are crucial to the rehabilitation process. By measuring shoulder joint mobility before rehabilitation, the goals and difficulty levels of the rehabilitation activities can be set according to the severity of the patient's condition, which is determined based on the maximum angle of shoulder joint motion when performing various rehabilitation movements, to accommodate each patient's rehabilitation needs. These measurements also allow patients to recognize the effects of rehabilitation, thereby increasing their motivation and compliance. Currently, measurements of shoulder joint mobility are conducted by therapists and using protractors. Along with the growth of telerehabilitation [9–11], there is increasing need for patients to measure shoulder joint mobility on their own at home in order to understand the progress of rehabilitation. However, it is difficult for patients to operate protractors alone at home.

With recent advancements in microelectromechanical systems, the size and cost of various types of sensors have declined considerably, leading to increased applications. Inertial measurement units (IMUs) are primarily used to measure the movement direction and orientation of a physical object. Incorporating wireless transmission technologies, the wireless IMU (WIMU) sensor was proposed as a wearable sensor and its applications in motor rehabilitation have increased [12, 13]. In this research, by integrating wireless IMU sensors with VR interactive technology, an innovative self-measurement system combined with a set of shoulder joint mobility exercises was developed, facilitating home-based objective assessments of patients' shoulder mobility. The research objectives of this study were as follows: (1) to combine wireless IMU sensors with VR interactive technology to produce an innovative shoulder joint mobility self-measurement system; (2) to verify the feasibility and accuracy of the developed system via clinical trials; and (3) to investigate patients' usage intentions for this system.

## 2. Methods

### 2.1. System Design

**Figure 1:**
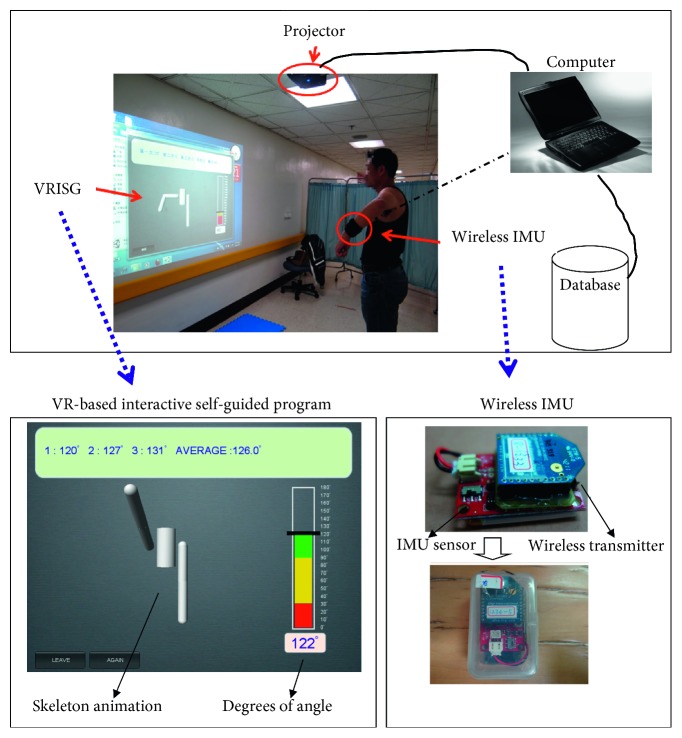
System architectures.

The two essential elements to define virtual reality are immersion and interaction. The system architecture comprised two units: a WIMU and a VR-based interactive self-guided program (VRISG). The system architecture design is shown in Figure 1. In our design, projector is used to display the virtual environment in order to provide the immersion. WIMU is applied that the user is able to interact with the virtual environment.

**Figure 2:**
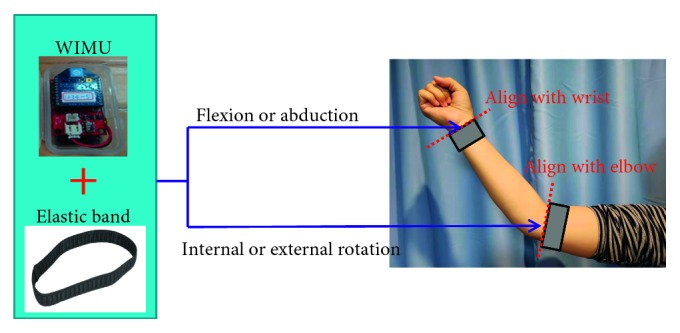
Physical setting of IMU.

The WIMU is primarily used to detect the shoulder joint position. Physically, the WIMU is attached to the user's affected side at wrist or elbow according to movement types, as shown in Figure 2. In addition, their performance of various shoulder joint mobility exercises could be assessed to determine the joint angle. Data of the shoulder joint angle and position are then transmitted wirelessly to the VRISG for processing. The proposed system comprised two parts: an IMU and a wireless transmission module (as shown in Figure 1). The IMU used in this study was a 9DOF Razor IMU. This IMU contains three sensor chips: triple-axis gyroscope, triple-axis accelerometer, and triple-axis magnetometer. Using these three sensor chips, the posture value (pitch, yaw, and row) of an object can be measured. After the IMU obtains posture data, the wireless transmission module XBee (manufactured by Digi International) transmits data signals to the XBee receiver connected to a computer. Finally, the game engine reads the signals and uses animations to present users' movement postures.

**Figure 3:**
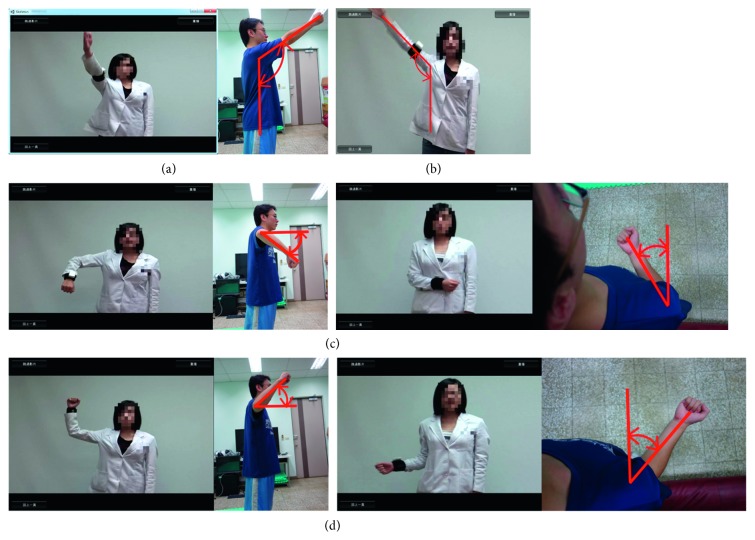
Movement types. (a) Flexion. (b) Abduction. (c) Internal rotation. (d) External rotation.

The VRISG uses the received data and animation functions to realistically simulate the human body. A projector is then used to project the simulated image onto a wall. The system uses a 3D Unity game engine and related software programs to produce a skeleton animation and user interface for measuring shoulder joint mobility when performing various movements. Corresponding activities were designed for self-guided measurement, with the movements including abduction, flexion, internal rotation, and external rotation, as shown in Figure 3. For measuring joint mobility during each movement, an instructional video is provided to assist users in understanding the correct way to perform each movement and the important steps they should be aware of during the measurement stage. Next, during the measurement process, the system guides users through skeleton animations based on shoulder joint angle measurements and presents user posture immediately as real-time visual feedback. This enables users to clearly understand the range and margin of their shoulder joint movements. Concurrently, an angle bar graph and angle value column appear on the right side of the computer screen and display shoulder joint angle measured by the IMU, informing users of their shoulder joint movement angle. Each movement should be performed three times, and the outcome is measured every time. Every movement cycle begins with users raising their upper limbs and gradually rotating the shoulder joint until the maximum movement angle is reached. The movement cycle ends when users relax their upper limbs. This movement cycle must be performed three times. The three angle measurements and the average of these measurements are displayed as a reference for patients and physical therapists. Furthermore, after each measurement is complete, the data are stored in a database to provide therapists with an understanding and analysis of each patient's rehabilitation status and progress.

### 2.2. Participants

The clinical trials conducted for this research were proactive, intervening, randomly assigned, and single blind. This study obtained consent for participation from 25 people, specifically, 10 men and 15 women, with an average age of 56.25 years. The average duration of the condition was 8.2 months. This study project was approved by the Institutional Review Board of Taipei Veterans General Hospital with VGHIRB: 2012-07-004A. The participant inclusion and exclusion criteria were as follows:

#### 2.2.1. Inclusion Criteria

1. At least 20 years of age
2. No previous experience of physical therapy
3. Normal cognition and able to follow the system use instructions
4. Clinical diagnosis of frozen shoulder
5. A signed consent form

#### 2.2.2. Exclusion Criteria

1. A history of injury, dislocation, or surgery to the shoulder or humerus
2. Prior treatment with hyaluronic acid injections in the shoulder
3. A history of cervical spondylotic radiculopathy or degenerative arthritis of the shoulder
4. A history of terminal malignant disease
5. Pregnancy

### 2.3. Experimental Procedure

**Figure 4:**
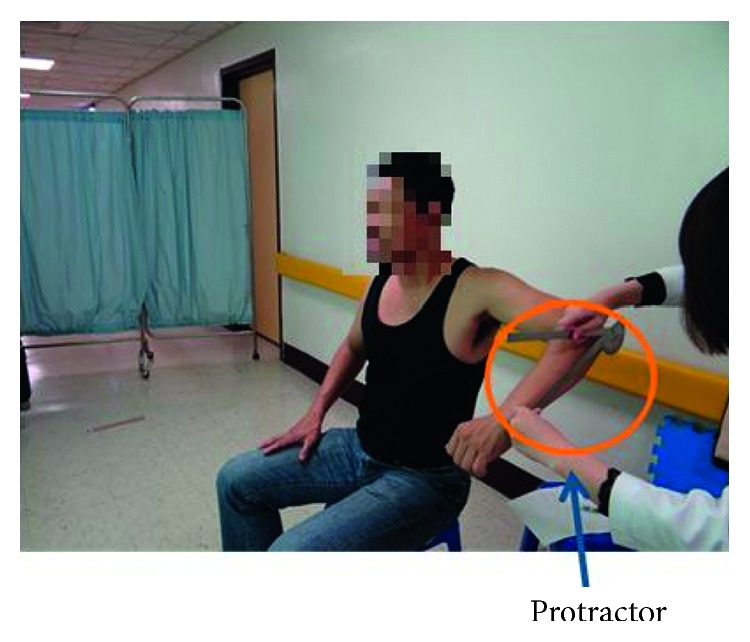
Using a protractor for measurement.

Before the experiment, the participants were required to sign an experiment consent form and understand the research procedures. The experiment primarily measured the patients' shoulder joint mobility. The movements that were performed in the experiment to measure shoulder joint mobility were divided into the following four categories: flexion, abduction, internal rotation, and external rotation. Two types of measurement methods were employed, which are as follows:

1. To use the innovative shoulder joint mobility self-measurement system proposed in this study for conducting measurements. During the experiment, the participants observed changes in the angle and mobility of their shoulder joint as well as the measurement results projected on the screen, as shown in Figure 1.
2. To use the traditional method, which involved physical therapists using a protractor for measurement, the patients were not provided any information regarding the measurements, as shown in Figure 4.

In this study, every participant was measured using both methods. During these measurements, they were required to perform the four shoulder joint mobility movements in sequence, and each movement was measured three times. Subsequently, the three measurements of each movement were averaged and recorded by a physical therapist. These data acquired from the first measurement method were classified as the experimental group, while data from the second method were classified as the control group. The experimental group and control group were measured separately by two physical therapists.

The participants in the experimental group first followed the sequence of the movements to attach the WIMU to the specified location and then used the VRISG to conduct measurements. Please refer to Section 2.2 for more details regarding the measurement process and content.

For the control group, a different physical therapist than the one assigned to the experimental group employed the traditional measurement method, where shoulder joint mobility was primarily measured by using traditional tools including a protractor. No software system was used to assist with the experimental process. The mobility measurement steps and movement sequence adopted for the control group were identical to those of the experimental group. Thus, the participants' shoulder joint mobility when performing each movement was measured three times by the physical therapist using a protractor combined with other traditional techniques. The measurement results were then averaged and recorded.

### 2.4. Measurement and Analysis

To verify the accuracy of the innovative shoulder joint mobility self-measurement system proposed in this study for clinical applications, the correlation and difference between the results of the experimental and control groups were further investigated by evaluating and comparing the analysis results of the Pearson's correlation coefficient and Wilcoxon signed-rank tests. The primary functions of a correlation coefficient are to identify the linear relationship between two random variables and to calculate the strength of their linear relationship. If the absolute value of the population correlation coefficient is near 1, the linear relationship between two variables increases in strength and correlation. The reason for conducting a Wilcoxon signed-rank test was because the experimental and control group samples were from the same population. Thus, this analysis method was adopted to investigate whether the difference between the results provided by the two methods was significant. Using the two aforementioned analysis methods to examine the correlation and difference between the results of the two measurement methods, the accuracy of the proposed system was verified.

## 3. Results

The first analysis method involved using correlation coefficients to compare the data provided by the two measurement methods; the analysis results are shown in Table 1. The results show that the calculated correlation coefficients were close to 1, and the *P* values all reached the level of significance. This indicates that the results of the two measurement methods possessed a high correlation.

For the second measurement method, a Wilcoxon signed-rank test was adopted to perform a paired-samples *t*-test analyzing the difference between the results provided by the two measurement methods. The analysis results are shown in Table 2. The *P* values calculated according to the paired-samples *t*-test results for the four movement measurements all exceeded 0.05. Thus, the measurement results obtained for the experimental group and the control group showed no significant differences.

## 4. Discussion

For this experiment, clinical trials were conducted on 25 patients. When performing self-measurements using the system proposed in this paper, the participants were able to follow the system instructions and measure their joint mobility themselves, verifying the feasibility of the proposed system.

According to the findings of the two analyses conducted, as mentioned previously, the measurements obtained using the proposed shoulder joint mobility self-measurement system and those obtained using a traditional protractor exhibited a high correlation and no significant differences. The similarity of the two measurement results verifies the accuracy of the proposed shoulder joint mobility self-measurement system.

The system proposed in this study comprises two aspects, software and hardware. Because of recent technological advancements, the size and weight of the wireless IMU sensor hardware have become substantially more compact and light. In addition, the precision of such hardware has increased while the cost decreased. Therefore, from a user perspective, acceptance of this system should be high and distribution should be fairly easy. The corresponding software program guides patients in completing self-assessments of shoulder joint mobility. This system not only exhibits technical accuracy and consistency, but also satisfies demands for user-centered designs. Furthermore, this system can be used to reduce the clinical burdens of therapists and extend the treatment into the patients' home, facilitating home-based healthcare.

According to the results of interviews conducted with therapists and patients, the therapists believe that the proposed system can effectively measure patients' joint mobility with reliable accuracy. In addition, the system can reduce the human resources and time required to assess patients' joint mobility using traditional methods. According to the observations of the therapists, patients can easily operate the system by themselves to measure their shoulder joint mobility. However, they asserted that should this system be used to replace therapists completely, side effects and issues, particularly social psychological issues, may result. Therefore, further investigations are required to find the optimum use strategy for this system and therapist assistance, for example, how patients should periodically undergo measurements by therapists at medical therapy centers in addition to using the system at home for self-measurements. The results of the patient interviews showed that the patients were confident and willing to operate the proposed system to enhance their understanding of their rehabilitation progress. However, they also acknowledged that compared with using the system at home for self-measurements, the psychological support and encouragement provided by therapists during traditional measurements and face-to-face assessments was highly valued.

## 5. Study Limitations

The primary limitations of this study resulted from the wireless IMU sensor hardware, namely, the size, weight, and “wearability” of the device, which influence people's willingness to use the system. The sensors' precision and wireless transmission speed can directly influence the sensitivity of interactions between the user and guidance system, which indirectly affects the user's perceptions. The guidance software designed for this system cannot provide patients with the psychological aspects of support and trust that a real therapist can.

## 6. Conclusions

In this study, an IMU sensor was successfully combined with interactive VR technology to develop an innovative shoulder joint mobility self-measurement system for patients diagnosed with frozen shoulder. Clinical trials were conducted with 25 patients; the results of which verified the feasibility of the system. The results of an analysis and comparison of the measurements provided by the proposed system and those obtained using traditional measurement methods show that a high correlation and no significant differences are exhibit between the two. This confirms that the proposed system can effectively and reliably measure patients' shoulder joint mobility. According to the results of interviews conducted with therapists and patients, the therapists believe that the system can effectively reduce the human resources and time required to assess patients' shoulder joint mobility while providing accurate and reliable results. The patients indicated that they were confident and willing to operate the system themselves to understand their rehabilitation progress.

In the future, additional novel MEMS technologies are expected to be employed to produce wireless IMU sensors that are comparatively smaller in size, lighter in weight, and higher in accuracy, thereby increasing the convenience of self-operated systems. Furthermore, large-scale clinical trials are set to be conducted to determine the reliability and validity of the proposed system. In the future, the application of advanced Internet technology and cloud technology combined with the concept of long-distance medical rehabilitation will popularize the system developed in this study for use at home.

## Figures and Tables

**Table 1:** Comparison of the correlation between the measurement results for the experimental and control groups.

Flexion	Abduction	External rotation	Internal rotation
0.997	0.978	0.897	0.984
*P*=*∗∗*	*P*=*∗∗*	*P*=*∗∗*	*P*=*∗∗*
Correlation level		Significance level = 0.05	
0 ≦ |*r*| ＜ 0.3: low		^*∗*^ *p* < 0.05	
0.3 ≦ |*r*| ＜ 0.7: medium		^*∗∗*^ *p* < 0.01	
0.7 ≦ |*r*| ＜ 1: high			

**Table 2:** Results of the Wilcoxon signed-rank test for the experimental and control group measurements.

	Flexion	Abduction	External rotation	Internal rotation
*P* value	0.556	0.129	1.044	2.547

Significance level = 0.05; ^*∗*^*p* < 0.05; ^*∗∗*^*p* < 0.01.

## Data Availability

The data used to support the findings of this study are available from the corresponding author upon request.
